# Minimally Invasive Surgery for the Excision and Repair of Cesarean Scar Defect: A Scoping Review of the Literature

**DOI:** 10.3390/medicina61071123

**Published:** 2025-06-21

**Authors:** Daniela Surico, Alessandro Vigone, Carlotta Monateri, Mario Tortora, Carmen Imma Aquino

**Affiliations:** 1 Department of Translational Medicine, Università del Piemonte Orientale, Gynecology and Obstetrics, AOU “Maggiore della Carità”, 28100 Novara, Italy; daniela.surico@med.uniupo.it (D.S.); alessandro.vigone@maggioreosp.novara.it (A.V.); carlotta.monateri@gmail.com (C.M.); 2 Neuroradiology Unit, Department of Advanced Biomedical Sciences, Università “Federico II”, 80138 Naples, Italy; mario.tortora@ymail.com

**Keywords:** isthmocele, cesarean scar defect, cesarean section complications, cesarean scar pregnancy, laparoscopy, robotic-assisted surgery

## Abstract

*Background and Objectives*: The isthmocele is a pouch-shaped defect in the anterior uterine wall, site of a previous cesarean section, due to a scar defect or dehiscence. The prevalence could be underestimated, but the rate of cesarean section is still high in the world. The preferable technique to correct this anomaly is not clearly indicated in the literature. Our objective is to evaluate the literature on the surgical treatment of isthmocele in pre-Cesarean women treated with minimally invasive technique. Our hypothesis is that robotic treatment is more effective than other procedures in women desirous of having children. *Materials and Methods*: The words “isthmocele”, “laparoscopy”, “robot” and “cesarean scar pregnancy” were searched on the main online scientific search sources (PubMed, Google Scholar, Scopus, WES, and Embase, etc.). We included articles in English and French, chosen for the relevance to the topic. We have decided to include also surgical corrections of isthmocele linked to pregnancies at the site of the defect, with particular attention to video training explanation. *Results*: We analyzed the literature about the minimally invasive surgery for the repair of an isthmocele, evaluating 20 articles. Comparing several surgical techniques, robotic-assisted laparoscopy could be an effective method to correct the defect, without high risk of intraoperative complications. *Conclusions*: As indicated in the literature, robotic tailored excision and repair of isthmocele (and of concomitant cesarean scar pregnancy) could be advantageous and safe, and it is necessary to promote video-training about this technique.

## 1. Introduction

The isthmocele is a pouch-shaped niche in the cesarean scar area on the anterior uterine wall, due to its defect or dehiscence. Most cases occur in women with one previous cesarean section [1]. Isthmocele could also be a diverticulum of the myometrial defect, like fibrous, vascularized tissue, lined with endometrium, with the possible collect of mucus and menstrual blood. The niche could have anechoic aspect in the lower uterine segment of at least 2.0 mm in depth, other studies evaluated also different cut-off [1]. A lower hysterotomy, the incomplete tissue closure, the anomalous adhesions of the uterine wall, and a genetic predisposition could contribute to the isthmocele. This situation was most often related to subfertility, miscarriage, but also ectopic pregnancy (cesarean scar pregnancy, CSP), placenta accreta spectrum problems, uterine dehiscence or rupture during pregnancy or at delivery, pelvic pain, and postmenstrual bleeding [1,2].

There are several hypotheses to explain the implications in infertility: an adverse environment for sperm transport and embryo implantation; the scar defect as a physical barrier; psychological problems in case of difficulties in conceive [1]. Transvaginal ultrasound (US) is a valid diagnostic tool: the shape and morphology could be different, as a round, square or wedge-shaped cavity, or even a cribriform area [3]. In fact, a hypoechoic image or a myometrial discontinuity of the wall could be highlighted in correspondence of the hysterotomy, with dimension >1 mm or a myometrial thinning > of 2 mm. In the US evaluation, it is important to consider defect size (three orthogonal planes) and volume dimension, minimum free myometrium or thickness of the residual myometrium, thickness of the adjacent myometrium, shape, and position or distance between isthmocele and uterine fundus and between internal uterine orifice and fundus. The diagnosis is mainly made through two-dimensional (2D) transvaginal ultrasound, with the eventual injection of contrast agents (i.e., hysterosalpingography SHG); three-dimensional (3D) US is a new tool that could be considered, but without significant diagnostic efficacy. Magnetic Resonance Imaging (MRI) could be reserved in complex cases [1,3]. There is no universally accepted classification of isthmocele; some categorizations are based on the size of the defect: large, when there is a myometrial reduction major than 50–80% of the wall thickness or with a minimal residual myometrium. The scar defect can also be sub-classified through multiple other ways:

- Volume Calculation (Base x Height))/2, as Simple or first degree < 15 mm^2^; Simple with one branch or second degree = 16–25 mm^2^ or Complex with more than one branch or third degree > 25 mm^2^.
- Deficiency Ratio, the ratio between residual myometrium and healthy myometrium x 100, as Large when the thickness of the residual myometrium is <50% of the healthy myometrium or Small when the thickness of the residual myometrium is >50% of the healthy myometrium.
- Measure of the minimum free myometrium, as Large if the residual myometrium is <2 mm with US, <2.5 mm with SHG, < 3 mm with MRI or Small, if the residual myometrium is >2 mm with US, > 2.5 mm with SHG, >3 mm with MRI.
- Shape (triangle, semicircle, rectangle, circular, drop, inclusion cyst) [2].

The diagnostic US image is the simple sagittal uterine plane: it is an easier and more acceptable approach than SHG and/or MRI [4]. Delphi consensuses regarding the standard possible criteria could be useful in daily practice for non-pregnant women or in the case of early scar pregnancy [4,5].

The literature is discordant about the best procedure to use for the treatment, but the relief of symptoms is described in most surgically treated patients, with scarce evidence regarding fertility and adequate procedure [2,6]. The technique to prefer must be considered among hysteroscopic, vaginal, laparotomic, laparoscopic approach, evaluating patient’s discomfort, desire for fertility, and the dimension of isthmocele. The excision with myometrium reconstruction may be considered if the residual tissue thickness is less than 3 mm [6,7,8]. In terms of outcomes, robot-assisted laparoscopic repair significantly improved myometrial thickness [6,9,10].

Our objective is to review the literature about minimally invasive surgical management of isthmocele, with a focus on robot- assisted laparoscopy. Our second objective is to spread the description of video reports, reviewing published articles that could help in training surgeons to learn the procedure.

## 2. Methods

### Relevant Sections

**Figure 1:**
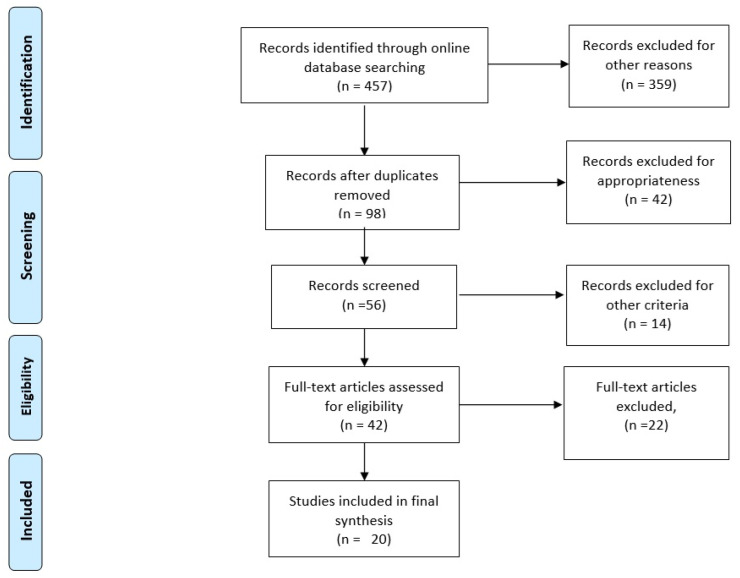
Inclusion flow-chart.

The Cochrane Systematic Review Database, MEDLINE, EMBASE, CINAHL, CENTRAL, and reference lists of well-known research from 2009 to 2025 were all thoroughly searched. For the same period, a gray literature search was also carried out using online databases. MeSH headings, when appropriate, were utilized in all literature searches. To make sure the search was accurate, an author examined it twice. The title and abstract screening included every search result. The words “isthmocele”, “cesarean scar pregnancy”AND “laparoscopy”,”robot”, NOT “laparotomy” OR “pharmacological” were searched on the main online scientific search sources, as indicated in our inclusion flow-chart (Figure 1).

After results from 457 articles were obtained, we excluded duplicates and not strictly scientifically related publications. We considered 56 studies, but finally included 20 articles published or in press in English and French, chosen for the relevance to the topic (Table 1). We decided to include surgical corrections of isthmocele linked to pregnancies at the site of the defect (CSP, cesarean scar pregnancy) to be able to compare the greatest number of experiences on the surgical technique. We preferred research with surgical video descriptions, because we are promoters of training through graphic support. We followed PRISMA guidelines for scoping review, and have registered it in the Open Science Framework (https://osf.io/43vmp/) accessed on 19 June 2025 [11].

## 3. Results

As evidenced (Table 1), the literature indicates the possibility of routinary use of minimally invasive surgical techniques (robot-assisted laparoscopy, concomitant use of hysteroscopy). All the articles in our review are based on the correction of isthmocele through laparoscopy, with robotic-assisted surgery [10,12,13,14,15,16,17,18,19,20,21,22,23,24,25,26,27,28,29,30]. Seven papers consider more than one patient in their description [10,14,18,20,21,23,29]. One study employed robotic surgery and carbon dioxide laser fiber, under hysteroscopy and near-infrared guidance [16]. Hysteroscopy was employed with laparoscopy in several papers [12,16,17,18,19,20,21,22,23,27,28]. Embolization and hemostatic clips were clearly evaluated as an additional surgical tool in three studies [15,20,30]. Of the 20 papers, Firefly fluorescent technology was used only in three case reports [12,16,18]. In the larger included case series (33 patients) [10], robotic surgery is described as a procedure managed by an expert surgeon. After the catheterization of the bladder, surgery was mainly performed through two trocars of 8 mm and one of 10 mm, plus an optical trocar. Vesico-uterine space was opened and dissected until the availability of the niche, reaching healthy tissue and removing the fibrotic area. The suture was executed in Monocryl 0 (poliglecaprone 25 suture, monofilament, Ethicon) or Vicryl 1 (polyglactin 910 suture, undyed braided, Ethicon). An US or MRI was performed three or six months after the treatment [10].

The aim of our review was to analyze the experience described in the literature about the minimally invasive robotic surgery for the correction of the isthmocele: the outcomes seem to be encouraging and in favor of a routinary use, when not contraindicated.

In fact, as the main results of the analyzed studies (Table 1), patients after robotic correction reported improving of symptoms and of quality of life, increasing in uterine wall thickness, satisfaction and resolution of infertility with successful treatment for all the patients. Twelve scientific works also considered a video-illustrated description of the procedure [12,13,15,16,17,19,22,23,24,25,26,27]: this is essential for our review, because we would like to provide clear reference to in-training or less expert surgeons.

## 4. Discussion

We evaluated the literature on the treatment of isthmocele in pre-Cesarean women treated with minimally invasive surgery: our hypothesis is that this treatment is more effective than other techniques in women desirous of having children.

Uterine scars can be related to hysterotomy after myomectomy and/or hysteroscopic surgery of congenital uterine malformations or cesarean section (CS) [31]. The rate of CS is stably increased, and this is related to several long-term negative sequela, with the persistence of the phenomenon also during Covid-19 pandemic [32,33]. Breech presentation is one of the most common anomalies of fetal presentation, and one of the most common causes of CS. Compared to vaginal delivery, the maternal morbidity rate for CS is almost three times higher. It is commonly acknowledged that CS poses greater dangers to mothers than vaginal birth [34]. Compared to patients experiencing vaginal delivery, these include increased blood loss, thrombotic events, unexpected hysterectomy, surgical damage to other organs, mortality, longer hospital stays with higher costs, and more readmissions. Fibrotic areas, persistent pain, and intestinal blockage brought on by adhesive disease are additional maternal consequences of CS. Furthermore, a prior cesarean delivery may increase the risk of placental anomalies, unexplained stillbirth, and, in many cases, repeated surgical delivery in subsequent pregnancies [34].

The scar defect may occur for the presence of one or more known risk factors, related to the increased tension in the lower uterine segment and a reduction in scar vascularization. Among the risk factors, those demonstrated are patient-related, as repeated cesarean section, retroverted uterus, high body mass index (BMI), hypertension, surgical factors, CS at cervical dilatation > 5 cm. Data on the incidence of isthmocele are conflictual, varying between 6.9% and 69% (in Italy, 24–84%): it occurs in approximately 60% of women after a primary CS and 100% after 3 CSs [35,36]. Uterine scar dehiscence does not include the visceral peritoneum, but during pregnancy the umbilical cord, fetus, and placenta could be encased within the uterus. The trial of labor post-cesarean is recommended with a vaginal delivery success rate between 60% and 77%. Interestingly, the success of delivery increases with an interval major than 6 months and inferior to 24 months, with no differences between type of uterine incision closure and delivery outcomes [34,35,36,37]. The correction of the isthmocele could be essential to avoid uterine dehiscence or rupture, that is, a full thickness tear of the wall including uterine serosa. It is a fatal complication that occurs with a frequency of 0.2% in women with previous CS and 2 cases in 10,000 overall maternities [37]. Isthmocele repair could diminish the miscarriage rate and other obstetric complications. The repair of the scar defect can also restore potential fertility and prevent sub-infertility. In fact, another consideration is about the increased diffusion of Assisted Reproductive Technology (ART) and the relatively augmented risk of ectopic pregnancy with CSP [38,39,40,41]. In fact, isthmocele also has a negative influence on live birth rate (LBR) in patients who underwent ART [42]. Post-embryo transfer gestations in women with a previous CS result in reduced biochemical pregnancy and LBR compared to women with previous vaginal delivery [43], and laparoscopic isthmocele treatment seemed to improve reproduction [44].

Medical care is possible for patients with abnormal genital bleeding, pain, and without a desire for fertility [6]. As shown in the literature, if residual uterine thickness is below 2.5–3 mm, surgical excision should be considered [45]. In fact, in symptomatic patients and with secondary infertility, surgery could be appropriate [46,47]. The available surgical procedures are hysteroscopy, laparotomy, laparoscopy (also robotic assisted), vaginal repair, and combined techniques [2,6,33,48]. Clinical practice considers different possibilities, validated depending on the initial indication [1]. Hysteroscopic technique could be the safest and most effective in case of adequate residual myometrial thickness; it is functional, quick, ambulatorial, and not expensive. It is not indicated in women with a desire for fertility due to the lack of effectiveness on myometrium thickness, and in cases of residual tissue over the defect < 3 mm, there is a higher risk of perforation and bladder injuries. Laparoscopic and vaginal approaches are preferred for thinner residual myometrium over the scar, and when hysteroscopy gave no results [6]. Hysteroscopy concomitant to laparoscopy with transillumination of the scar defect allow to find and easily intervene on the isthmocele, with an accurate excision of the edges though the direct infrared fluorescence imaging in real time [49] and may also be transposed to near-infrared laparoscopy, with increased success in the case of irregular situations [50].

The main limitations include a dislocated surgical view in cases of hysterotomy, a fixed uterus, or narrow vaginal access (in case of vaginal surgery); the use of general anesthesia; and the expensive costs of laparoscopy and robotic-assisted surgery [2,6].

In general, these procedures are safe, functional, non-invasive, and may restore anatomy in case of large defects. Surgery for the treatment of symptomatic isthmocele improves bleeding symptoms in more than 80% of women. Minor evidence is described to improve fertility or reduce obstetric complications in asymptomatic patients [51]. Globally, robotic-assisted laparoscopy plus hysteroscopy with near-infrared fluorescence and Firefly technology could be the preferable surgical approaches in most cases [6,12,29,46].

Limitations are mainly related to the heterogeneity of information and to the sample size described for each included article. The strength of our review is the updated and complete analysis of the topic, with a particular focus on graphical support. To spread the procedure, learning by surgeons and resident medical doctors must take place through revision papers—like our review—and through the dissemination of training videos, considering the previous published research [12,16,17,18,19,21,22,23,27,28,52,53].

## 5. Conclusions

Since surgery was found to alleviate bleeding symptoms in most patients, we found sufficient evidence to justify robotic treatment for symptomatic isthmocele.

In cases of patients with sufficient residual myometrial thickness overlaying the isthmocele, hysteroscopic treatment of the condition may be the safest and most successful procedure. When hysteroscopic treatment is inconclusive and individuals have a thinner remaining myometrium above the defect (<2.5 mm), vaginal approach and laparoscopic robotic surgery may be the best options. Vaginal access must be considered in situations such as lower dislocation of the isthmocele, sliding uterus, and absence of pelvic comorbidities. A laparoscopic or robotic-assisted procedure can be used in the case of women with fertility desire or large defects suffering symptoms and the failure of other treatments. Considering this knowledge, it is important to spread the use of the different types of minimally invasive surgery, particularly robotic techniques, to successfully treat isthmocele and improve patients’ quality of life.

## Figures and Tables

**Table 1:** Review of the literature about correction of isthmocele.

Authors/Year and Type of Study	Sample and Medical Indication	Surgical Technique and Anesthesia	Gestational Age (if CSP), in Weeks	Description of the Procedure in the Article	Duration (MOT)	Results
Surico et al. [12], 2025. Video-case report	1 patient, Isthmocele	Intraoperative hysteroscopy and a robotic-assisted laparoscopy with Firefly technology. General anesthesia	/	Cold scissors were used to remove the isthmocele entirely. To heal the myometrium, a double-layer interrupted Vicryl 2-0 suture was used. There were no intraoperative difficulties.	90 min	Correction of the defect after 30 days following surgery, healthy pregnancy after 14 months of the surgery.
van Reesema et al. [13], 2025. Video-case report	1 patient, Isthmocele and CSP	Robot-assisted laparoscopy. General anesthesia	6	The tissue around the CSP was injected with 12 mL of diluted vasopressin (20 units vasopressin in 100 mL normal saline). The cervicovaginal junction was then marked by inserting an end-to-end anastomosis sizer into the patient’s vagina. The cervix was reapproximated using two layers of running 2-0 V-loc sutures on the anterior and posterior cervix, followed by modified mattress stitches at the bilateral apices using two distinct 2-0 V-loc sutures (Medtronic, Minneapolis, MN).	N.A.	Correction of the defect and negative saline-infused sonography.
Muendane et al. [14], 2025. Cohort study-	24 patients, Isthmocele	Robotic-assisted laparoscopic niche repair preceded by diaphanoscopy. General anesthesia	/	Vicryl 2-0 continuous non-locked suture was used for the serosal layer of the three-layer closure, while running and barbed suture (V-Loc™ 3-0) were used for the two inner layers, which contained the first half of the myometrium without endometrium, and a second outer myometrial layer. Slow-resorbable polydioxanone sutures (PDS 2-0) are used to stitch the round ligaments to the anterior uterine wall. This reduces tension on the scar and allows for an antefexed uterine position for two to three months while the wound heals.	145 min	Widths and depths of the niches were significantly reduced (*p* < 0.001), with increment of the pregnancy rate.
Cardaillac et al. [10], 2023. Cohort study	33 patients, Isthmocele	Robot-assisted laparoscopic surgery. General anesthesia	/	Monopolar scissors, bipolar forceps, and gripping forceps were the tools utilized. The whole fibrotic tissue of the isthmocele was removed. The operator used either Vicryl 1 (polyglactin 910 suture, undyed braided, Ethicon) or Monocryl 0 (poliglecaprone 25 suture, undyed monofilament, Ethicon) X stitches to suture the wound. Three or six months following the procedure, an MRI or ultrasound was conducted.	98 min	Improved myometrial thickness [mean difference 2.71; 95% CI, 1.91–3.51], *p* = 0.0005); improved pregnancy rate and symptoms. 51.5% of the patients stated that they would choose to have this surgery again.
Huang et al. [15], 2023. Video-case report	1 patient, Isthmocele	Ethylene vinyl alcohol copolymer (EVAC) embolization followed by robot-assisted laparoscopic technique. General anesthesia	/	EVAC image guided uterine embolization. Isthmocele and residual EVAC in the cavity are identified hysteroscopically,and defined by fluorescence transillumination. Creation of a bladder flaps and dissection of the retroperitoneal region to skeletonize uterine arteries. Vascular clamps are used to temporarily block the uterine arteries to reduce operative blood loss. The isthmocele is removed, and any remaining intracavitary EVAC is eliminated. The uterine blood flow was restored through multilayer, bidirectional hysterotomy closure and vascular clamp removal.	N.A.	Successful treatment.
Walker et al. [16], 2023. Video-case report-	1 patient, Isthmocele	Robot-assisted laparoscopic surgery, employing a carbon dioxide laser fiber, under hysteroscopy and Firefly near-infrared guidance. General anesthesia	/	Five units of vasopressin were injected into the uterus. Energy source for dissection was a 5-watt carbon dioxide laser fiber (Lumenis FiberLase). A 20-watt laser was used for the excision in the infrared/gray scale, eliminating the whole region that the Firefly had marked. A two-layer closure was used for hysterotomy: a running 2-0 PDS Stratafix (Ethicon) was placed after four mattress sutures of 2-0 Vicryl (Ethicon). The peritoneal layer was closed in a running way with 2-0 PDS Stratafix (Ethicon). A 14 French Malecot catheter (Bard) was inserted into the uterus cavity. After covering the repair region with interceed adhesion barrier (Gynecare), the process was completed.	N.A.	Improved myometrial thickness.
Yoon R et al. [17], 2021. Video- case report	1 patient, Isthmocele and CSP	Robot-assisted laparoscopic surgery and hysteroscopy. General anesthesia	First trimester	Diluted vasopressin, carefully applying electrosurgical energy and, a multilayer closure were used.	N.A.	Successful treatment.
Hoffmann et al. [18], 2021. Case series	5 patients, Isthmocele and CSP	Combined medical and surgical approach with robotic assisted laparoscopic resection, Firefly, and hysteroscopy. General anesthesia	6–8	Adesiolisis and a bladder flap were performed. The ectopic pregnancy was cut off from the uterine defect using monopolar instruments. The uterine defect was subsequently transversely repaired utilizing a two-layer closure with #0 barbed suture in a continuous, non-locked method. Chromopertubation was carried out.	N.A.	Valid treatment of current ectopic pregnancy and repair of uterine defect.
Katebi Kashi et al. [19], 2021. Video-case report	1 patient, Isthmocele and CSP	Robotic assisted excision and hysteroscopy with metroplasty. General anesthesia	6	Following the failure of methotrexate therapy, the patient had a simple robotic aided excision of the CSP and a two-layer metroplasty: Step 1: Make a bladder flap; Step 2: Separate and remove CSP; Step 3: Close the hysterotomy in two layers; and Step 4: Perform a hysteroscopy.	N.A.	Valid treatment of current ectopic pregnancy and repair of uterine defect, followed by a successful pregnancy.
Hofgaard et al. [20], 2021. Case series	14 patients, Isthmocele and CSP	Robot-assisted laparoscopy and intraoperative occlusion of the uterine blood supply. General anesthesia	6–13	After the pelvic sidewalls were dissected, nine women had their distal internal iliac arteries clipped with detachable metal clips (Bulldog clips; Aesculap, Tuttlingen, Germany), then taken off. The myometrium surrounding the defect was removed and then readjusted in two layers using either a continuous V-Loc suture or a single 2-0 Vicryl (Ethicon, Norderstedt, Germany). The myometrium surrounding the CSP was injected with one to three units of Vasopressin (Pitressin^®^; Link Pharmaceuticals Ltd., Auckland, New Zealand).	N.A.	Uneventful surgeries, 64% of patients conceived naturally.
Wang et al. [21], 2021. Case series-	20 patients: robotic (n = 3) or conventional laparoscopic (n = 17) surgery, Isthmocele and CSP	Robotic or laparoscopic repair, and hysteroscopy. General anesthesia	N.A.	The myometrium surrounding the cervix was penetrated with diluted vasopressin. The bladder was occasionally carefully separated from the cervix using a monopolar scissor. The uterine sound (TY90, TAIYU Inc., Tokyo, Japan) was then bent to around 90 degrees using a 2 cm tip. Whole-layer sutures with barbed threads were used to close the myometrium.	137 min	Improvement in postmenstrual vaginal bleeding, decrease in the depth and width of the cesarean scar defect, and increased residual myometrial thickness.
Nyangoh Timoh et al. [22], 2020. Video- case report	1 patient, Isthmocele	Robotic-assisted laparoscopy using hysteroscopy treatment. General anesthesia	N.A.	The vesico-uterine cleft was treated as initial stage. To obtain healthy tissue, they ultimately removed the tissue scars from the isthmocele. Additionally, a slow absorption suture of the various regions of the uterus was applied.	120 min	Successful treatment.
Guan et al. [23], 2020. Video-case report	2 patients, Isthmocele	Robotic single-site laparoscopy and hysteroscopy. General anesthesia	N.A.	The fascia was grabbed and penetrated with Mayo scissors. The bladder was carefully removed from the lower uterine portion and then backfilled. Cold scissors (Endoshears) were used to trim the edges, and in the second surgery, a monopolar hook. Two layers of V-Loc suture were used to close the uterine defect. An extra V-Loc suture was used to seal the peritoneum in a running pattern.	90 and 85 min (Respectively, for the two patients)	Successful repair of the cesarean scar defect and resolution of the patients’ symptoms.
Ye et al. [24], 2020. Video-case report	1 patient, Isthmocele and CSP	Robot-assisted laparoscopic surgery. General anesthesia	7	Surgery was used to repair the hysterotomy and remove the ectopic pregnancy through a robot-assisted laparoscopic procedure.	N.A.	Effective procedure, no more symptoms.
Schmitt et al. [25] 2017. Video-case report	1 patient, Isthmocele and residual CSP	Robot-assisted laparoscopic surgery. General anesthesia	N.A.	Video step-by-step explanation of the procedure trough minimally invasive surgery.	N.A.	Valid correction of the pouch, possibility to treat residual cesarean scar pregnancy.
Siedhoff et al. [26], 2015. Video Case report	1 patient, Isthmocele and residual CSP	Robot-assisted laparoscopic surgery. General anesthesia	N.A.	The defect was made visible by opening the vesicovaginal area. Diluted vasopressin was administered. Pregnancy tissue and scar were removed. A delayed-absorbable barbed suture was used to seal the hysterotomy, extending the procedure from laparoscopic myomectomy. The first layer was imbricated with a second.	N.A.	Correction of the defect, no more symptoms, the woman became pregnant.
Mahmoud et al. [27], 2015. Video Case report	1 patient, Isthmocele	Hysteroscopy and robotic-assisted laparoscopic repair. General anesthesia	/	Executed a bladder flap, the scar tissue around the defect was dissected, and the area closed with delayed absorbable suture. Chromopertubation confirmed the watertightness.	N.A.	Regular normal periods and negative hysterosalpingogram.
La Rosa et al. [28], 2013. Review and case report	1 patient, Isthmocele	Robot-assisted laparoscopic surgery and hysteroscopy. General anesthesia	/	Adhesiolysis was carried out utilizing electrocautery and sharp dissection. An incision was made in the uterus. Unipolar electrocautery was used to refresh the defect’s edges. Two layers of 0-Vicryl sutures were used to seal the uterus.	N.A.	Successful repair.
Yalcinkaya et al. [29], 2011. Cases report	2 patients, Isthmocele	Robotic-assisted laparoscopic surgery. General anesthesia	/	Using 45 Watts’ coagulating current and electrode, midline adhesions were taken down between the omentum and abdominal wall, until cervicouterine fascia was clearly detected. Using a hook electrode, the edges were trimmed off. Hemostasis was obtained and the retracted visceral peritoneum was carefully approximated to the pubo-cervico-vesicular fascia at midline to partially cover the peritoneal defect. Vitagel was instilled into the sub-peritoneal region to promote hemostasis.	N.A.	No more symptoms, women became pregnant.
Persson et al. [30], 2009. Case report	1 patient, Isthmocele and CSP	Robot-assisted laparoscopic surgery with temporary occlusion of the main uterine blood supply. General anesthesia	11	After methotrexate and mifepristone treatments in previous days, the pelvic side walls were surgically dissected and the distal internal iliac arteries were clipped with detachable metal clips (bulldog clips; Aesculap, Tuttlingen, Germany). The propria ligaments were clamped similarly. Using a laparoscopic retrieval bag and a suction device, the pregnancy was removed. The myometrium surrounding the defect was treated using a single 2-0 Vicryl suture (Ethicon, Norderstedt, Germany). The metal clips were finally taken off.	171 min	Feasible and safe technique for a cesarean scar pregnancy and for the correction of the isthmocele.

CSP = cesarean scar pregnancy, N.A. = not available, MOT = mean operation time, min = minutes.

## Data Availability

The original contributions presented in this study are included in the article. Further inquiries can be directed to the corresponding author.
